# [13,27-Dichloro-3,6,9,17,20,23-hexa­azatetracyclo­[23.3.1.1^11,15^.0^2,6^]triaconta-1(29),9,11,13,15(30),16,23,25,27-nona­ene-29,30-diol-κ^5^ *N* ^17^,*N* ^20^,*N* ^23^,*O* ^29^,*O* ^30^]bis­(nitrato-κ^2^ *O*,*O*′)europium(III) nitrate methanol hemisolvate

**DOI:** 10.1107/S1600536809013440

**Published:** 2009-05-07

**Authors:** Xia-Li Yue

**Affiliations:** a Department of Chemistry, Huazhong Agricultural University, Wuhan, Hubei 430070, People’s Republic of China

## Abstract

The title compound, [Eu^III^(NO_3_)_2_(C_24_H_28_Cl_2_N_6_O_2_)]NO_3_·0.5CH_3_OH, is isostructural with the Gd^III^ and Ho^III^ complexes of the analogous macrocyclic ligand, with both Cl atoms replaced by methyl groups. The Eu atom exhibits a nine-coordinate distorted tricapped trigonal-prismatic coordination geometry. The methanol solvent mol­ecule is disordered about a twofold rotation axis with occupancies of 0.543 (12):0.457 (12).

## Related literature

For applications of macrocyclic lanthanide complexes, see: Alexander (1995 ▶); Bunzli & Piguet (2002 ▶). For related structures, see: Hu, Chen *et al*. (2007 ▶); Hu, Qiu, Yuan & Pan (2007 ▶); Hu, Qiu, Zhao & Pan (2007 ▶).
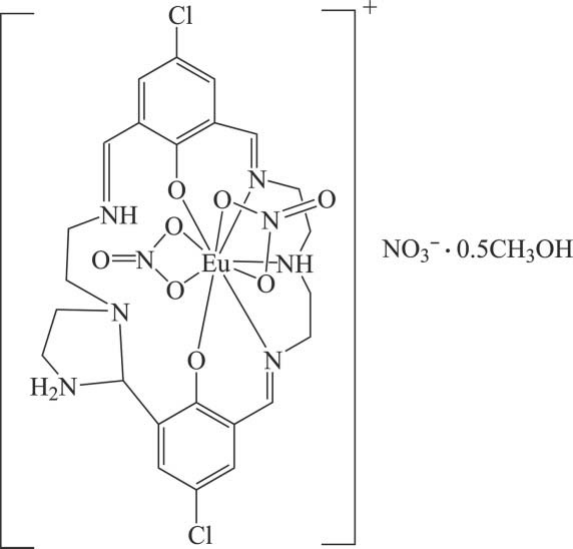


## Experimental

### 

#### Crystal data

[Eu(NO_3_)_2_(C_24_H_28_Cl_2_N_6_O_2_)]NO_3_·0.5CH_4_O
*M* *_r_* = 857.44
Monoclinic, 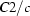

*a* = 23.7371 (16) Å
*b* = 14.3327 (10) Å
*c* = 19.3880 (13) Å
β = 91.804 (1)°
*V* = 6592.9 (8) Å^3^
*Z* = 8
Mo *K*α radiation
μ = 2.14 mm^−1^
*T* = 293 K
0.30 × 0.22 × 0.20 mm

#### Data collection

Bruker SMART CCD diffractometer
Absorption correction: multi-scan (*SADABS*; Bruker, 2000 ▶) *T*_min_ = 0.58, *T*_max_ = 0.66
18638 measured reflections
6469 independent reflections
5337 reflections with *I* > 2σ(*I*)
*R*_int_ = 0.016

#### Refinement

*R*[*F* ^2^ > 2σ(*F* ^2^)] = 0.032
*wR*(*F* ^2^) = 0.088
*S* = 1.02
6469 reflections
453 parameters
62 restraints
H atoms treated by a mixture of independent and constrained refinement
Δρ_max_ = 1.37 e Å^−3^
Δρ_min_ = −0.81 e Å^−3^

### 

Data collection: *SMART* (Bruker, 2000 ▶); cell refinement: *SAINT* (Bruker, 2000 ▶); data reduction: *SAINT*; program(s) used to solve structure: *SHELXTL* (Sheldrick, 2008 ▶); program(s) used to refine structure: *SHELXTL*; molecular graphics: *SHELXTL*; software used to prepare material for publication: *SHELXTL*.

## Supplementary Material

Crystal structure: contains datablocks global, I. DOI: 10.1107/S1600536809013440/bi2359sup1.cif

Structure factors: contains datablocks I. DOI: 10.1107/S1600536809013440/bi2359Isup2.hkl

Additional supplementary materials:  crystallographic information; 3D view; checkCIF report

## Figures and Tables

**Fig. 1.**
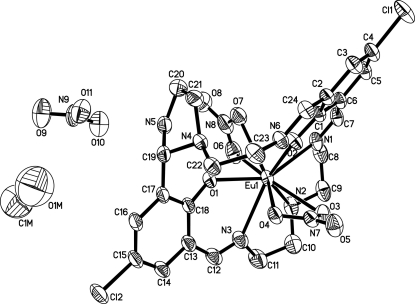
Molecular strucure with displacement ellipsoids drawn at the 30% probability level. H atoms are omitted.

**Fig. 2.**
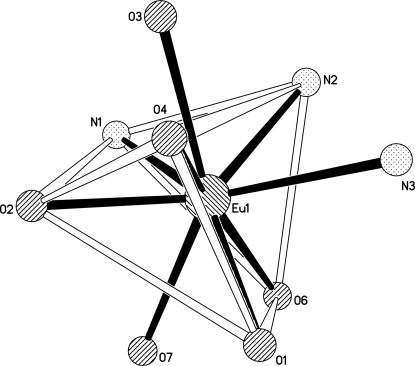
Coordination polyhedron around the EuIII atom.

## supplementary crystallographic information

### Comment

Lanthanide macrocyclic complexes have received attention on account of their many valuable applications, for example as fluorescent probes in biological systems and as new luminescent materials (Bunzli & Piguet, 2002; Alexander, 1995). Generally, the synthesis of lanthanide macrocyclic complexes is carried out by one-step condensation in the presence of a suitable lanthanide ion which acts as a template for the macrocycle formation.

Recently, Hu *et al.* have reported the crystal structures of Gd^III^, Ho^III^ and Lu^III^ complexes with the macrocyclic ligand derived from 2,6-diformyl-4-methylphenol and 1,5-diamino-3-azapentane (Hu, Chen, Qiu & Pan, 2007; Hu, Qiu, Yuan & Pan, 2007; Hu, Qiu, Zhao & Pan, 2007). Herein we report a new Eu^III^ analogue, synthesized by the same method using 2,6-diformyl-4-chlorophenol instead of 2,6-diformyl-4-methylphenol. The compound is isostructural with the previously reported Gd^III^ and Ho^III^ complexes.

### Experimental

1,5-Diamino-3-azapentane (1 mmol) was added dropwise to a methanolic solution (20 ml) of 2,6-diformyl-4-chlorophenol (1 mmol) and Eu(NO_3_)_3_.6H_2_O (0.5 mmol). After refluxing for 5 h, the solvent was removed and the resulting yellow solid was recrystallized from CH_3_CN to yield yellow block crystals.

### Refinement

All carbon-bound H atoms were generated geometrically (C—H = 0.93–0.97 Å) and included in the refinement as riding with *U*_iso_(H) = 1.2 or 1.5*U*_eq_(C). The H atoms of the N—H group and methanol molecule were located in difference Fourier maps. The former was refined freely, while the latter was constrained to ride on the O atom with *U*_iso_(H) = 1.5*U*_eq_(O). Atoms O10 and O11 in the uncoordinated nitrate and O8 in the coordinate nitrate are modelled as disordered. The N—O distances were restrained to be comparable within the two disorder components (with s.u. 0.005 Å) and the displacement parameters of the disordered atoms were restrained to approximate isotropic behaviour. The C—O distance of the methanol molecule was restrained to 1.40 (1) Å.

### Crystal data

**Table:**

[Eu(NO_3_)_2_(C_24_H_28_Cl_2_N_6_O_2_)]NO_3_·0.5CH_4_O	*F*(000) = 3432
*M**_r_* = 857.44	*D*_x_ = 1.728 Mg m^−^^3^
Monoclinic, *C*2/*c*	Mo *K*α radiation, λ = 0.71073 Å
Hall symbol: -C 2yc	Cell parameters from 8071 reflections
*a* = 23.7371 (16) Å	θ = 2.6–27.9°
*b* = 14.3327 (10) Å	µ = 2.14 mm^−^^1^
*c* = 19.3880 (13) Å	*T* = 293 K
β = 91.804 (1)°	Block, yellow
*V* = 6592.9 (8) Å^3^	0.30 × 0.22 × 0.20 mm
*Z* = 8	

### Data collection

**Table:**

Bruker SMART CCD diffractometer	6469 independent reflections
Radiation source: sealed tube	5337 reflections with *I* > 2σ(*I*)
graphite	*R*_int_ = 0.016
φ and ω scans	θ_max_ = 26.0°, θ_min_ = 2.0°
Absorption correction: multi-scan (*SADABS*; Bruker, 2000)	*h* = −28→29
*T*_min_ = 0.58, *T*_max_ = 0.66	*k* = −13→17
18638 measured reflections	*l* = −16→23

### Refinement

**Table:**

Refinement on *F*^2^	Primary atom site location: structure-invariant direct methods
Least-squares matrix: full	Secondary atom site location: difference Fourier map
*R*[*F*^2^ > 2σ(*F*^2^)] = 0.032	Hydrogen site location: inferred from neighbouring sites
*wR*(*F*^2^) = 0.088	H atoms treated by a mixture of independent and constrained refinement
*S* = 1.02	*w* = 1/[σ^2^(*F*_o_^2^) + (0.0424*P*)^2^ + 14.6115*P*] where *P* = (*F*_o_^2^ + 2*F*_c_^2^)/3
6469 reflections	(Δ/σ)_max_ = 0.001
453 parameters	Δρ_max_ = 1.37 e Å^−^^3^
62 restraints	Δρ_min_ = −0.81 e Å^−^^3^

### Special details

**Table:**

Geometry. All e.s.d.'s (except the e.s.d. in the dihedral angle between two l.s. planes) are estimated using the full covariance matrix. The cell e.s.d.'s are taken into account individually in the estimation of e.s.d.'s in distances, angles and torsion angles; correlations between e.s.d.'s in cell parameters are only used when they are defined by crystal symmetry. An approximate (isotropic) treatment of cell e.s.d.'s is used for estimating e.s.d.'s involving l.s. planes.
Refinement. Refinement of *F*^2^ against ALL reflections. The weighted *R*-factor *wR* and goodness of fit *S* are based on *F*^2^, conventional *R*-factors *R* are based on *F*, with *F* set to zero for negative *F*^2^. The threshold expression of *F*^2^ > σ(*F*^2^) is used only for calculating *R*-factors(gt) *etc*. and is not relevant to the choice of reflections for refinement. *R*-factors based on *F*^2^ are statistically about twice as large as those based on *F*, and *R*- factors based on ALL data will be even larger.

### Fractional atomic coordinates and isotropic or equivalent isotropic displacement parameters (Å^2^)

**Table:**

	*x*	*y*	*z*	*U*_iso_*/*U*_eq_	Occ. (<1)
Eu1	0.132267 (7)	0.505891 (12)	0.114103 (11)	0.04946 (8)	
Cl1	−0.09862 (5)	0.87749 (10)	0.19253 (8)	0.0924 (4)	
Cl2	0.44821 (4)	0.56791 (11)	0.04485 (7)	0.0868 (4)	
N1	0.02753 (14)	0.4948 (2)	0.1406 (2)	0.0578 (9)	
N2	0.08955 (15)	0.3511 (3)	0.0744 (2)	0.0720 (11)	
H2	0.1009	0.3075	0.1060	0.086*	
N3	0.20067 (15)	0.3992 (3)	0.0512 (2)	0.0682 (10)	
N4	0.24192 (12)	0.7536 (2)	0.16799 (16)	0.0532 (8)	
N5	0.25996 (14)	0.6549 (3)	0.25521 (17)	0.0677 (10)	
H5A	0.2367	0.6078	0.2429	0.081*	
H5B	0.2859	0.6335	0.2862	0.081*	
N6	0.14960 (12)	0.8091 (2)	0.09364 (17)	0.0500 (7)	
H6	0.1482 (19)	0.750 (4)	0.093 (2)	0.075 (15)*	
O1	0.22095 (10)	0.5406 (2)	0.15231 (14)	0.0581 (7)	
O2	0.09634 (10)	0.65382 (18)	0.12070 (15)	0.0543 (6)	
C1	0.05213 (14)	0.7012 (3)	0.13456 (18)	0.0448 (8)	
C2	0.05371 (14)	0.7998 (3)	0.13119 (19)	0.0479 (8)	
C3	0.00702 (16)	0.8538 (3)	0.1483 (2)	0.0576 (10)	
H3	0.0087	0.9186	0.1459	0.069*	
C4	−0.04138 (16)	0.8101 (3)	0.1685 (2)	0.0602 (10)	
C5	−0.04490 (15)	0.7141 (3)	0.1701 (2)	0.0584 (10)	
H5	−0.0783	0.6861	0.1831	0.070*	
C6	0.00017 (14)	0.6583 (3)	0.15280 (19)	0.0497 (9)	
C7	−0.00850 (15)	0.5575 (3)	0.1519 (2)	0.0576 (10)	
H7	−0.0447	0.5370	0.1607	0.069*	
C8	0.00742 (19)	0.3978 (3)	0.1386 (3)	0.0761 (14)	
H8A	−0.0334	0.3967	0.1393	0.091*	
H8B	0.0221	0.3642	0.1787	0.091*	
C9	0.02697 (18)	0.3523 (3)	0.0740 (3)	0.0811 (15)	
H9A	0.0127	0.2889	0.0712	0.097*	
H9B	0.0126	0.3864	0.0340	0.097*	
C10	0.1133 (2)	0.3240 (4)	0.0092 (3)	0.0850 (15)	
H10A	0.1036	0.3703	−0.0257	0.102*	
H10B	0.0974	0.2647	−0.0056	0.102*	
C11	0.1762 (2)	0.3157 (4)	0.0168 (3)	0.0911 (17)	
H11A	0.1858	0.2606	0.0437	0.109*	
H11B	0.1921	0.3087	−0.0284	0.109*	
C12	0.25325 (19)	0.4135 (3)	0.0422 (3)	0.0697 (12)	
H12	0.2708	0.3721	0.0128	0.084*	
C13	0.28854 (17)	0.4860 (3)	0.0720 (2)	0.0586 (10)	
C14	0.34386 (19)	0.4933 (3)	0.0490 (2)	0.0660 (12)	
H14	0.3567	0.4506	0.0170	0.079*	
C15	0.37933 (16)	0.5622 (3)	0.0730 (2)	0.0625 (11)	
C16	0.36114 (15)	0.6265 (3)	0.1197 (2)	0.0582 (10)	
H16	0.3852	0.6741	0.1348	0.070*	
C17	0.30710 (14)	0.6210 (3)	0.1445 (2)	0.0542 (9)	
C18	0.27015 (14)	0.5485 (3)	0.1232 (2)	0.0520 (9)	
C19	0.28796 (15)	0.6941 (3)	0.1932 (2)	0.0573 (10)	
H19	0.3201	0.7329	0.2081	0.069*	
C20	0.2276 (2)	0.7336 (4)	0.2848 (2)	0.0845 (16)	
H20A	0.2458	0.7561	0.3271	0.101*	
H20B	0.1894	0.7146	0.2943	0.101*	
C21	0.22782 (18)	0.8080 (4)	0.2290 (2)	0.0702 (13)	
H21A	0.1912	0.8374	0.2232	0.084*	
H21B	0.2560	0.8555	0.2392	0.084*	
C22	0.25275 (15)	0.8039 (3)	0.1050 (2)	0.0584 (10)	
H22A	0.2638	0.7600	0.0698	0.070*	
H22B	0.2839	0.8468	0.1134	0.070*	
C23	0.20158 (16)	0.8584 (3)	0.0787 (2)	0.0608 (10)	
H23A	0.2013	0.9193	0.1004	0.073*	
H23B	0.2038	0.8674	0.0293	0.073*	
C24	0.10390 (15)	0.8487 (3)	0.1123 (2)	0.0517 (9)	
H24	0.1032	0.9136	0.1137	0.062*	
N7	0.12585 (13)	0.6082 (3)	−0.01481 (18)	0.0570 (8)	
O3	0.09247 (12)	0.5410 (2)	−0.00393 (16)	0.0666 (7)	
O4	0.17132 (10)	0.6095 (2)	0.02117 (15)	0.0602 (7)	
O5	0.11402 (13)	0.6698 (3)	−0.05529 (18)	0.0798 (9)	
N8	0.14009 (18)	0.4670 (5)	0.2623 (3)	0.0941 (16)	
O6	0.13883 (15)	0.4020 (3)	0.2184 (2)	0.0917 (12)	
O7	0.13077 (13)	0.5480 (3)	0.24185 (17)	0.0784 (9)	
O8	0.1559 (4)	0.4690 (9)	0.3256 (3)	0.099 (2)	0.543 (12)
O8'	0.1383 (5)	0.4232 (9)	0.3188 (5)	0.099 (2)	0.457 (12)
N9	0.36911 (18)	0.6104 (3)	0.3621 (2)	0.0765 (11)	
O9	0.4091 (2)	0.5910 (4)	0.3993 (3)	0.1301 (16)	
O10	0.3339 (3)	0.5519 (5)	0.3452 (4)	0.104 (2)	0.687 (7)
O11	0.3656 (3)	0.6888 (3)	0.3356 (3)	0.0957 (17)	0.687 (7)
O10'	0.3531 (7)	0.5516 (10)	0.3194 (7)	0.104 (2)	0.313 (7)
O11'	0.3356 (5)	0.6730 (8)	0.3750 (7)	0.0957 (17)	0.313 (7)
C1M	0.5000	0.6209 (14)	0.2500	0.220 (8)	
H1MA	0.5386	0.6246	0.2370	0.330*	0.50
H1MB	0.4985	0.6041	0.2978	0.330*	0.50
H1MC	0.4808	0.5744	0.2224	0.330*	0.50
O1M	0.4733 (8)	0.7024 (13)	0.2367 (13)	0.295 (11)	0.50
H1WD	0.4442	0.7310	0.2458	0.442*	0.50

### Atomic displacement parameters (Å^2^)

**Table:**

	*U*^11^	*U*^22^	*U*^33^	*U*^12^	*U*^13^	*U*^23^
Eu1	0.03538 (11)	0.04471 (12)	0.06810 (15)	0.00163 (7)	−0.00116 (8)	0.00535 (8)
Cl1	0.0600 (7)	0.0939 (9)	0.1253 (11)	0.0351 (6)	0.0364 (7)	0.0337 (8)
Cl2	0.0409 (5)	0.1263 (11)	0.0944 (9)	0.0189 (6)	0.0194 (5)	0.0119 (8)
N1	0.0387 (17)	0.0527 (19)	0.082 (2)	−0.0089 (14)	−0.0029 (16)	0.0151 (16)
N2	0.055 (2)	0.052 (2)	0.108 (3)	0.0004 (16)	−0.014 (2)	0.002 (2)
N3	0.059 (2)	0.055 (2)	0.091 (3)	0.0117 (17)	−0.0009 (19)	−0.0088 (18)
N4	0.0346 (15)	0.069 (2)	0.0562 (19)	−0.0009 (14)	0.0057 (13)	−0.0043 (15)
N5	0.0447 (18)	0.104 (3)	0.054 (2)	0.0002 (18)	−0.0010 (15)	0.0061 (19)
N6	0.0378 (15)	0.0477 (19)	0.065 (2)	−0.0017 (14)	0.0060 (14)	0.0057 (15)
O1	0.0344 (13)	0.0762 (19)	0.0637 (17)	0.0021 (12)	0.0033 (11)	−0.0021 (14)
O2	0.0368 (13)	0.0453 (14)	0.0814 (19)	0.0022 (11)	0.0128 (12)	0.0044 (13)
C1	0.0346 (16)	0.051 (2)	0.049 (2)	−0.0001 (14)	0.0021 (14)	0.0074 (15)
C2	0.0407 (18)	0.050 (2)	0.053 (2)	0.0026 (15)	0.0044 (15)	0.0061 (16)
C3	0.051 (2)	0.055 (2)	0.067 (3)	0.0102 (18)	0.0099 (18)	0.0108 (19)
C4	0.0405 (19)	0.071 (3)	0.070 (3)	0.0138 (18)	0.0092 (18)	0.016 (2)
C5	0.0366 (18)	0.072 (3)	0.067 (3)	0.0045 (18)	0.0066 (17)	0.020 (2)
C6	0.0334 (17)	0.060 (2)	0.056 (2)	−0.0001 (15)	0.0029 (15)	0.0144 (17)
C7	0.0327 (18)	0.065 (3)	0.076 (3)	−0.0073 (17)	0.0001 (17)	0.019 (2)
C8	0.050 (2)	0.058 (3)	0.120 (4)	−0.010 (2)	−0.003 (2)	0.021 (3)
C9	0.054 (2)	0.053 (3)	0.134 (5)	−0.008 (2)	−0.018 (3)	−0.003 (3)
C10	0.077 (3)	0.062 (3)	0.115 (4)	0.003 (2)	−0.013 (3)	−0.023 (3)
C11	0.077 (3)	0.063 (3)	0.132 (5)	0.009 (3)	0.000 (3)	−0.032 (3)
C12	0.061 (3)	0.060 (3)	0.088 (3)	0.022 (2)	0.005 (2)	−0.007 (2)
C13	0.046 (2)	0.062 (3)	0.068 (3)	0.0165 (18)	0.0005 (18)	0.0042 (19)
C14	0.051 (2)	0.080 (3)	0.068 (3)	0.030 (2)	0.006 (2)	0.005 (2)
C15	0.0349 (19)	0.085 (3)	0.068 (3)	0.017 (2)	0.0078 (18)	0.013 (2)
C16	0.0341 (18)	0.077 (3)	0.063 (2)	0.0057 (18)	0.0001 (16)	0.012 (2)
C17	0.0325 (17)	0.077 (3)	0.053 (2)	0.0058 (17)	−0.0008 (15)	0.0065 (19)
C18	0.0350 (17)	0.067 (2)	0.054 (2)	0.0120 (17)	0.0006 (15)	0.0077 (18)
C19	0.0350 (18)	0.084 (3)	0.053 (2)	−0.0041 (18)	−0.0018 (16)	−0.001 (2)
C20	0.063 (3)	0.131 (5)	0.061 (3)	0.010 (3)	0.010 (2)	−0.012 (3)
C21	0.048 (2)	0.093 (3)	0.071 (3)	−0.005 (2)	0.011 (2)	−0.022 (2)
C22	0.0402 (19)	0.068 (3)	0.068 (3)	−0.0038 (18)	0.0108 (18)	0.001 (2)
C23	0.048 (2)	0.051 (2)	0.084 (3)	−0.0066 (17)	0.015 (2)	0.007 (2)
C24	0.045 (2)	0.045 (2)	0.065 (2)	0.0000 (16)	0.0040 (17)	0.0054 (17)
N7	0.0444 (18)	0.062 (2)	0.065 (2)	0.0023 (15)	0.0068 (15)	0.0017 (17)
O3	0.0598 (17)	0.0610 (17)	0.078 (2)	−0.0098 (15)	−0.0092 (14)	0.0052 (15)
O4	0.0374 (13)	0.0713 (19)	0.0721 (18)	0.0014 (12)	0.0036 (12)	0.0033 (14)
O5	0.0617 (18)	0.089 (2)	0.088 (2)	−0.0007 (17)	−0.0002 (16)	0.0328 (19)
N8	0.058 (2)	0.149 (5)	0.076 (3)	0.031 (3)	0.017 (2)	0.045 (3)
O6	0.067 (2)	0.087 (3)	0.120 (3)	0.0060 (19)	0.000 (2)	0.043 (2)
O7	0.0608 (19)	0.105 (3)	0.070 (2)	0.0129 (19)	0.0101 (15)	0.015 (2)
O8	0.106 (3)	0.104 (4)	0.087 (3)	0.011 (3)	0.002 (2)	0.020 (3)
O8'	0.106 (3)	0.104 (4)	0.087 (3)	0.011 (3)	0.002 (2)	0.020 (3)
N9	0.074 (3)	0.065 (2)	0.089 (3)	0.005 (2)	−0.027 (2)	0.016 (2)
O9	0.110 (3)	0.139 (3)	0.138 (3)	−0.006 (3)	−0.044 (3)	0.035 (3)
O10	0.098 (3)	0.094 (3)	0.120 (4)	−0.015 (3)	−0.017 (3)	0.029 (3)
O11	0.100 (3)	0.082 (3)	0.103 (3)	−0.002 (2)	−0.026 (2)	0.003 (2)
O10'	0.098 (3)	0.094 (3)	0.120 (4)	−0.015 (3)	−0.017 (3)	0.029 (3)
O11'	0.100 (3)	0.082 (3)	0.103 (3)	−0.002 (2)	−0.026 (2)	0.003 (2)
C1M	0.218 (9)	0.221 (9)	0.222 (9)	0.000	0.009 (5)	0.000
O1M	0.294 (12)	0.290 (12)	0.300 (12)	−0.013 (5)	0.001 (5)	0.000 (5)

### Geometric parameters (Å, °)

**Table:**

Eu1—O1	2.265 (2)	C10—H10A	0.970
Eu1—O2	2.290 (2)	C10—H10B	0.970
Eu1—O3	2.499 (3)	C11—H11A	0.970
Eu1—O4	2.533 (3)	C11—H11B	0.970
Eu1—O6	2.513 (4)	C12—C13	1.444 (6)
Eu1—O7	2.551 (3)	C12—H12	0.930
Eu1—N1	2.559 (3)	C13—C14	1.403 (6)
Eu1—N2	2.549 (4)	C13—C18	1.417 (6)
Eu1—N3	2.566 (4)	C14—C15	1.370 (7)
Cl1—C4	1.742 (4)	C14—H14	0.930
Cl2—C15	1.742 (4)	C15—C16	1.371 (6)
N1—C7	1.264 (5)	C16—C17	1.387 (5)
N1—C8	1.470 (5)	C16—H16	0.930
N2—C10	1.452 (7)	C17—C18	1.413 (6)
N2—C9	1.485 (5)	C17—C19	1.490 (6)
N2—H2	0.910	C19—H19	0.980
N3—C12	1.282 (6)	C20—C21	1.519 (7)
N3—C11	1.480 (6)	C20—H20A	0.970
N4—C22	1.448 (5)	C20—H20B	0.970
N4—C19	1.459 (5)	C21—H21A	0.970
N4—C21	1.465 (5)	C21—H21B	0.970
N5—C20	1.489 (6)	C22—C23	1.518 (6)
N5—C19	1.501 (5)	C22—H22A	0.970
N5—H5A	0.900	C22—H22B	0.970
N5—H5B	0.900	C23—H23A	0.970
N6—C24	1.286 (5)	C23—H23B	0.970
N6—C23	1.459 (5)	C24—H24	0.930
N6—H6	0.85 (5)	N7—O5	1.209 (4)
O1—C18	1.318 (4)	N7—O4	1.267 (4)
O2—C1	1.286 (4)	N7—O3	1.269 (4)
C1—C2	1.414 (5)	N8—O7	1.245 (7)
C1—C6	1.433 (5)	N8—O6	1.262 (7)
C2—C3	1.401 (5)	N8—O8'	1.265 (7)
C2—C24	1.440 (5)	N8—O8	1.273 (6)
C3—C4	1.376 (5)	N9—O9	1.206 (5)
C3—H3	0.930	N9—O10	1.221 (4)
C4—C5	1.380 (6)	N9—O11'	1.230 (5)
C5—C6	1.385 (5)	N9—O10'	1.233 (5)
C5—H5	0.930	N9—O11	1.238 (4)
C6—C7	1.459 (6)	C1M—O1M^i^	1.351 (10)
C7—H7	0.930	C1M—O1M	1.351 (10)
C8—C9	1.499 (7)	C1M—H1MA	0.960
C8—H8A	0.970	C1M—H1MB	0.960
C8—H8B	0.970	C1M—H1MC	0.960
C9—H9A	0.970	O1M—O1M^i^	1.35 (4)
C9—H9B	0.970	O1M—H1WD	0.826
C10—C11	1.501 (7)		
			
O1—Eu1—O2	97.01 (10)	C8—C9—H9B	109.7
O1—Eu1—O3	125.16 (10)	H9A—C9—H9B	108.2
O2—Eu1—O3	74.57 (10)	N2—C10—C11	110.4 (4)
O1—Eu1—O6	80.42 (11)	N2—C10—H10A	109.6
O2—Eu1—O6	121.17 (14)	C11—C10—H10A	109.6
O3—Eu1—O6	150.28 (13)	N2—C10—H10B	109.6
O1—Eu1—O4	75.31 (9)	C11—C10—H10B	109.6
O2—Eu1—O4	69.04 (9)	H10A—C10—H10B	108.1
O3—Eu1—O4	50.72 (9)	N3—C11—C10	110.8 (4)
O6—Eu1—O4	154.86 (10)	N3—C11—H11A	109.5
O1—Eu1—N2	130.32 (11)	C10—C11—H11A	109.5
O2—Eu1—N2	132.66 (10)	N3—C11—H11B	109.5
O3—Eu1—N2	76.33 (12)	C10—C11—H11B	109.5
O6—Eu1—N2	74.98 (15)	H11A—C11—H11B	108.1
O4—Eu1—N2	116.53 (12)	N3—C12—C13	128.0 (4)
O1—Eu1—O7	70.82 (10)	N3—C12—H12	116.0
O2—Eu1—O7	73.14 (11)	C13—C12—H12	116.0
O3—Eu1—O7	145.58 (11)	C14—C13—C18	119.2 (4)
O6—Eu1—O7	50.30 (14)	C14—C13—C12	117.6 (4)
O4—Eu1—O7	124.64 (11)	C18—C13—C12	123.2 (4)
N2—Eu1—O7	118.80 (13)	C15—C14—C13	121.1 (4)
O1—Eu1—N1	147.85 (11)	C15—C14—H14	119.4
O2—Eu1—N1	71.36 (9)	C13—C14—H14	119.4
O3—Eu1—N1	81.65 (11)	C14—C15—C16	120.4 (4)
O6—Eu1—N1	80.66 (11)	C14—C15—Cl2	120.0 (4)
O4—Eu1—N1	123.97 (9)	C16—C15—Cl2	119.6 (4)
N2—Eu1—N1	68.09 (12)	C15—C16—C17	120.3 (4)
O7—Eu1—N1	77.08 (11)	C15—C16—H16	119.8
O1—Eu1—N3	72.00 (11)	C17—C16—H16	119.8
O2—Eu1—N3	145.13 (11)	C16—C17—C18	120.8 (4)
O3—Eu1—N3	84.94 (11)	C16—C17—C19	118.9 (4)
O6—Eu1—N3	90.28 (14)	C18—C17—C19	120.2 (3)
O4—Eu1—N3	76.13 (11)	O1—C18—C17	119.2 (4)
N2—Eu1—N3	65.76 (12)	O1—C18—C13	122.9 (4)
O7—Eu1—N3	129.10 (12)	C17—C18—C13	117.9 (3)
N1—Eu1—N3	133.76 (11)	N4—C19—C17	116.0 (3)
C7—N1—C8	117.1 (3)	N4—C19—N5	98.1 (3)
C7—N1—Eu1	131.1 (3)	C17—C19—N5	113.3 (4)
C8—N1—Eu1	111.7 (3)	N4—C19—H19	109.6
C10—N2—C9	114.4 (4)	C17—C19—H19	109.6
C10—N2—Eu1	109.5 (3)	N5—C19—H19	109.6
C9—N2—Eu1	112.3 (3)	N5—C20—C21	104.2 (3)
C10—N2—H2	106.7	N5—C20—H20A	110.9
C9—N2—H2	106.7	C21—C20—H20A	110.9
Eu1—N2—H2	106.7	N5—C20—H20B	110.9
C12—N3—C11	115.9 (4)	C21—C20—H20B	110.9
C12—N3—Eu1	127.1 (3)	H20A—C20—H20B	108.9
C11—N3—Eu1	116.8 (3)	N4—C21—C20	102.0 (4)
C22—N4—C19	115.0 (3)	N4—C21—H21A	111.4
C22—N4—C21	117.9 (4)	C20—C21—H21A	111.4
C19—N4—C21	103.2 (3)	N4—C21—H21B	111.4
C20—N5—C19	105.8 (4)	C20—C21—H21B	111.4
C20—N5—H5A	110.6	H21A—C21—H21B	109.2
C19—N5—H5A	110.6	N4—C22—C23	112.2 (3)
C20—N5—H5B	110.6	N4—C22—H22A	109.2
C19—N5—H5B	110.6	C23—C22—H22A	109.2
H5A—N5—H5B	108.7	N4—C22—H22B	109.2
C24—N6—C23	124.6 (4)	C23—C22—H22B	109.2
C24—N6—H6	114 (3)	H22A—C22—H22B	107.9
C23—N6—H6	121 (3)	N6—C23—C22	110.9 (3)
C18—O1—Eu1	134.9 (2)	N6—C23—H23A	109.5
C1—O2—Eu1	144.0 (2)	C22—C23—H23A	109.5
O2—C1—C2	119.7 (3)	N6—C23—H23B	109.5
O2—C1—C6	122.6 (3)	C22—C23—H23B	109.5
C2—C1—C6	117.7 (3)	H23A—C23—H23B	108.0
C3—C2—C1	121.3 (3)	N6—C24—C2	124.6 (4)
C3—C2—C24	117.3 (3)	N6—C24—H24	117.7
C1—C2—C24	121.4 (3)	C2—C24—H24	117.7
C4—C3—C2	119.3 (4)	O5—N7—O4	121.6 (4)
C4—C3—H3	120.3	O5—N7—O3	121.9 (3)
C2—C3—H3	120.3	O4—N7—O3	116.4 (3)
C3—C4—C5	120.8 (4)	N7—O3—Eu1	94.9 (2)
C3—C4—Cl1	119.3 (3)	N7—O4—Eu1	93.4 (2)
C5—C4—Cl1	119.9 (3)	O7—N8—O6	118.3 (4)
C4—C5—C6	121.5 (4)	O7—N8—O8'	136.8 (8)
C4—C5—H5	119.2	O6—N8—O8'	102.5 (9)
C6—C5—H5	119.2	O7—N8—O8	109.3 (8)
C5—C6—C1	119.3 (4)	O6—N8—O8	131.8 (7)
C5—C6—C7	117.7 (3)	N8—O6—Eu1	96.0 (3)
C1—C6—C7	123.0 (3)	N8—O7—Eu1	94.6 (3)
N1—C7—C6	127.6 (3)	O9—N9—O10	121.4 (5)
N1—C7—H7	116.2	O9—N9—O11'	123.3 (8)
C6—C7—H7	116.2	O9—N9—O10'	117.6 (10)
N1—C8—C9	109.0 (4)	O11'—N9—O10'	116.6 (11)
N1—C8—H8A	109.9	O9—N9—O11	119.9 (5)
C9—C8—H8A	109.9	O10—N9—O11	118.4 (5)
N1—C8—H8B	109.9	O1M—C1M—H1MA	110.4
C9—C8—H8B	109.9	O1M—C1M—H1MB	111.7
H8A—C8—H8B	108.3	H1MA—C1M—H1MB	109.5
N2—C9—C8	109.6 (4)	O1M—C1M—H1MC	106.3
N2—C9—H9A	109.7	H1MA—C1M—H1MC	109.5
C8—C9—H9A	109.7	H1MB—C1M—H1MC	109.5
N2—C9—H9B	109.7		

Symmetry codes: (i) −*x*+1, *y*, −*z*+1/2.
